# Investigation of Magnetic Circular Dichroism Spectra of Semiconductor Quantum Rods and Quantum Dot-in-Rods

**DOI:** 10.3390/nano10061059

**Published:** 2020-05-30

**Authors:** Farrukh Safin, Vladimir Maslov, Yulia Gromova, Ivan Korsakov, Ekaterina Kolesova, Aliaksei Dubavik, Sergei Cherevkov, Yurii K. Gun’ko

**Affiliations:** 1 School of Photonics, ITMO University, 197101 St. Petersburg, Russia; maslov04@bk.ru (V.M.); korsakovivan@yandex.ru (I.K.); e.p.kolesova@gmail.com (E.K.); adubavik@corp.ifmo.ru (A.D.); S.cherevkov@gmail.com (S.C.); 2 School of Chemistry, Trinity College, Dublin 2 Dublin, Ireland; yulia.a.gromova@gmail.com (Y.G.); IGOUNKO@tcd.ie (Y.K.G.)

**Keywords:** magnetic circular dichroism, quantum nanocrystals, quantum rods, Dot-in-Rods

## Abstract

Anisotropic quantum nanostructures have attracted a lot of attention due to their unique properties and a range of potential applications. Magnetic circular dichroism (MCD) spectra of semiconductor CdSe/ZnS Quantum Rods and CdSe/CdS Dot-in-Rods have been studied. Positions of four electronic transitions were determined by data fitting. MCD spectra were analyzed in the ***A*** and ***B*** terms, which characterize the splitting and mixing of states. Effective values of ***A*** and ***B*** terms were determined for each transition. A relatively high value of the ***B*** term is noted, which is most likely associated with the anisotropy of quantum rods.

## 1. Introduction

Anisotropic colloidal semiconductor nanocrystals of various shapes have attracted a lot of attention over recent years due to their unique properties and potential applications. Quantum Rods (QRs) and Dot-in-Rods (DiRs) nanocrystals engage great interest in many fields of science and technology [1,2,3,4,5,6,7,8,9,10,11,12]. Due to their elongated shape, QRs and DiRs have high anisotropy in contrast to spherical quantum dots (QDs). Modern synthesis methods allow us to produce nanocrystals with high chemical and photostability. Careful engineering of the nanocrystal shell reduces blinking and increase a luminescence quantum yield up to 87% [1,2,3]. Today, quite accurate control of the QDs and DiRs parameters (core size, thickness, shell size) enables us to adjust the radiation wavelength, structure morphology, and light polarization parameters [4,5,6,7,8,9]. Unique optical properties together with mechanical stability and biocompatibility make QRs and DiRs good candidates for potential applications in photovoltaics, laser technology, and biomedical applications [10,11,12].

The study of the electronic structure of nanoparticles is usually carried out by optical spectroscopy. In particular, absorption spectroscopy is used very often. However, this technique does not always give complete information about electronic transitions in quantum nanostructures. Higher energy transitions are frequently overlapped and cannot be resolved in absorbance spectra. More detailed information on electronic transitions can be obtained by using magnetic circular dichroism spectroscopy (MCD) [13,14].

MCD is the differential absorption of left and right circularly polarized light (LCP and RCP), in the sample placed in a magnetic field oriented parallel to the direction of light propagation. MCD spectra provide information on the degeneracy and symmetry of electronic states and the paramagnetic properties of the systems under study. There are three main mechanisms which could induce changes in electronic transitions in the MCD spectra: (1) Zeeman splitting of degenerate excited states (***A***-term); (2) the effect of mixing of the zero-field states in the presence of magnetic field (***B***-term); (3) the difference in the populations of sublevels of an initially degenerate ground state (***C***-term). The ***A*** term appears in the MCD spectrum as a feature with the distinctive first derivative band shape. The ***A*** term without an admixture of the ***B*** and ***C*** terms and without overlapping with other transitions gives the position of the zero-field transition as the point of MCD spectrum intersection with the abscissa axis. The ***A*** term occurs in systems with a symmetry axis of the third or higher order. In a system with lower symmetry, only the ***B*** terms are present. The ***C*** term is observed only in systems with a degenerate ground state. The ***C*** term is normally negligible at room temperature [15].

For the first time, the MCD spectra of CdSe QDs were reported in 1998 [16]. The authors experimentally determined the effective g-factor for two lowest-energy exciton states. Both transitions had an ***A***-term shape. In contrast, MCD spectra of AgInS_2_/ZnS quantum dots include both ***A*** and ***B*** terms due to the anisotropy of the AgInS_2_ crystal lattice. MCD spectra of anisotropic CdSe nanoplates also demonstrate both ***A*** and ***B*** terms [17]. Moreover, the authors demonstrated that the combination of MCD and traditional absorption spectroscopy helps to specify the position of the poorly resolved electron transitions in spectra of nanostructures [17,18], but there are only a very limited number of publications on the investigation of quantum-size exciton transitions by the MCD method, and studies of anisotropic QRs or DiRs by this method have not been previously reported.

In this work, for the first time, we present the studies of the electronic transitions in CdSe/ZnS QRs and CdSe/CdS DiRs by MCD spectroscopy. The values of the ***A*** and ***B*** terms were determined for all absorption bands observed in the spectra.

## 2. Materials and Methods

We used CdSe/ZnS colloidal QRs with a diameter of 5 nm and a length of 35 nm coated with trioctylphosphine oxide (TOPO) synthesized by the hot injection method [19]. TOPO (Sigma Aldrich Inc, Darmstadt, Germany) coated CdSe/CdS DiRs were synthesized by the method described in previous publications [20]. Rods had a diameter of 6 nm, a length of 47 nm; QDs had a diameter of 3 nm. SEM images of QRs and DiRs are shown in Figure S3 in the Supplementary Information. Nanocrystals were synthesized by co-authors: S.C. and A.D. in our laboratory.

### 2.1. Measurement Procedure

A colloidal solution of QRs and DiRs in chloroform (Vekton, Russia) was prepared to measure the MCD and absorbance spectra. MCD and absorbance spectra were measured on a Jasco J-1500 circular dichroism spectrophotometer (Tokyo, Japan) equipped with electromagnetic unit MCD-581 at a field strength of ±1.5 T at room temperature.

### 2.2. MCD Spectra Analysis Technique

The MCD spectrum was described by Equation (1) [13,15]:

(1) $$\frac{\Delta D_{MCD}\left(E\right)}{E}=\gamma \left\{A_{1}\left(-\frac{df\left(E\right)}{dE}\right)+\left(B_{0}+\frac{C_{0}}{kT}\right)f\left(E\right)\right\}\left(\beta_{B}H\right)cz$$

$\Delta D_{MCD}\left(E\right)$—MCD spectrum; E = hν is photon energy; $A_{1}$, $B_{0}$, $C_{0}$—parameters determining the contributions of the terms ***A***, ***B***, ***C*** (at room temperature, $\frac{C_{0}}{kT}$ is usually negligible); f(E)—normalized absorption curve in the region of the investigated transition $\int_{0}^{\infty }f\left(E\right)dE=1$; k—Boltzmann constant; T—temperature; $\beta_{B}$—Bohr magneton; H—magnetic field strength; γ—constant proportional to the oscillator strength; c—sample concentration; z—optical path.

The expression describing the absorption spectrum $D_{L,R}^{0}\left(E\right)$ in the absence of a magnetic field, in these notations, was presented as:

(2) $$\frac{D_{L,R}^{0}\left(E\right)}{E}=\gamma D_{0}f\left(E\right)cz$$

where $D_{0}$ is the transition characteristic equal to the square of the transition dipole moment and measured in Debye^2^.

To estimate the numerical values of $A_{1}$, $B_{0}$ one usually uses the relative values *A*_1_/*D*_0_ and *B*_0_*/D*_0_. To determine these parameters from the experimental spectra, we used formulas [18] obtained from Equations (1) and (2) under the assumption that the absorption spectrum is described by a Gaussian curve:

(3) $$\frac{A_{1}}{D_{0}}=\frac{e^{1/2}}{2\times 2.35\beta_{B}}\frac{\left(Dif\right)\Gamma }{D_{m}H}$$

(4) $$\frac{B_{0}}{D_{0}}=\frac{1}{\beta_{B}}\frac{\Delta D_{m}}{D_{m}H}$$

where Γ is the full width at half maximum (FWHM) of the absorption band in cm^−1^, $D_{m}$ is the absorbance peak value in absorbance units, $\Delta D_{m}$ is the value at the maximum (or minimum) of the MCD transition also in absorbance units, (Dif) is the difference between the maximum (minimum) on the long-wavelength slope of the MCD transition and in the minimum (maximum) on the short-wavelength decline of this transition; H—magnetic field strength in Tesla, $\beta_{B}$ = 0.4671 cm^−1^/T.

### 2.3. Description of the Fitting Procedure

Firstly, positions of the electronic transitions were determined as the minima of the second derivative of the absorption spectrum. Further, MCD spectra were approximated by a linear combination of a Gaussian and its derivative: $MCD\left(\lambda \right)=ae^{-b{\left(\lambda -\lambda_{0}\right)}^{2}}+c\left(\lambda -\lambda_{0}\right)e^{-b{\left(\lambda -\lambda_{0}\right)}^{2}}$, where the first summand corresponds to the ***B*** term and the second one represents the ***A*** term, the spectrum is approximated as a sum of these expressions over all transitions. The parameter b depends on band FWHM: $b=4ln2/\Gamma^{2}$; the parameters a and c are proportional to the band amplitudes; λ—is wavelength; $\lambda_{0}$—is the center of the band. Band centers were fixed at the same wavelength as transitions in absorbance spectra. The a, b, c parameters were fitted in PeakFit software incorporated in the program «Origin 8» (OriginLab Corp.).

Next, the absorption spectra were approximated by the sum of Gaussians, with fixed FWHM and positions of the transitions determined during fitting of the MCD spectra. An extra Gaussian function with a maximum in the UV spectral region was added to simulate an underlying continuum of unresolved transitions, increasing towards short wavelengths [21,22].

Results of MCD and absorbance fitting were used for *A*_1_/*D*_0_ and *B*_0_/*D*_0_ calculation by Equations (3) and (4). It must be emphasized that in this approach, each band in the absorption spectrum is associated with only one transition in the MCD spectrum. In fact, each observed MCD transition consists of a group of closely spaced electronic transitions [16]. Therefore, calculated ***A*** and ***B*** terms can be considered only as some effective, summed values.

## 3. Results

**Figure 1:**
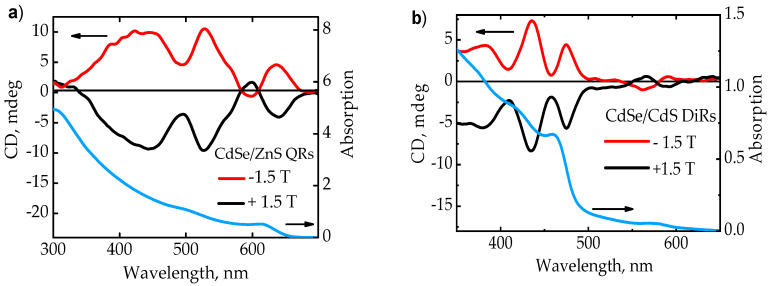
Magnetic circular dichroism (MCD) spectra and absorption spectra at a field strength of ±1.5 T: (**a**) CdSe/ZnS QRs: the red curve shows the MCD spectrum at a field strength of −1.5 T, the black curve shows the MCD spectrum at a field strength of +1.5 T; (**b**) CdSe/CdS DiRs: the red curve is the MCD spectrum at a field strength of −1.5 T, the black curve is the MCD spectrum at a field strength of +1.5 T.

Figure 1 shows the MCD and the absorption spectra of QRs and DiRs. In Figure 1a, the absorption spectra of CdSe/ZnS QRs clearly show the exciton transitions in the range of 475–650 nm. The MCD spectra were examined in detail including the region up to 400 nm. However, the consideration and assignment of the MCD spectra in the region of shorter wavelengths is a difficult task due to a large number of overlapping transitions. In Figure 1b, according to the absorption spectrum of CdSe/CdS DiRs, one can distinguish two regions: (i) CdSe QD absorption at 550–600 nm; (ii) CdS rod absorption with two noticeable bands at 400–450 nm, and 375–400 nm. For both samples, exact positions of transitions were determined as minima of the second derivation of absorbance spectra. More information on fitting the absorption spectra of QRs and DiRs is provided in the Supplementary Information.

**Figure 2:**
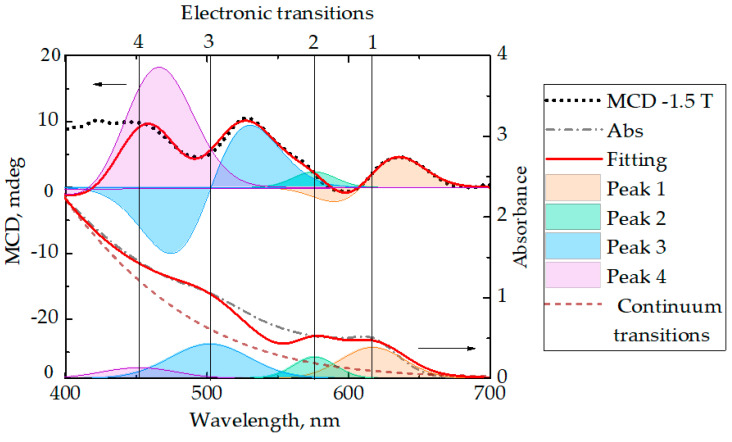
Fitting of MCD (top) and absorption spectrum (bottom) of QRs CdSe/ZnS. On the MCD spectrum the orange curve is the 1st band, the green curve is the 2nd band, the blue curve is the 3rd band, the violet curve is the 4th band, the dotted line is the QR CdSe/ZnS MCD spectrum at the field strength of −1.5 T, the orange curve is an approximation.

QRs MCD spectrum fitting was carried out in the range from 400 to 700 nm, the chi-square criterion (X^2^) was 1.096. Figure 2 demonstrates the results of MCD spectra fitting. Asymmetry of almost all fitted bands indicates the contribution of both the ***A*** and ***B*** terms to each transition. However, it should be noted that the 2nd band is Gaussian, which indicates the predominant contribution of the ***B*** term. The transition positions and corresponding numerical values of *A*_1_/*D*_0_ and *B*_0_/*D*_0_ in the QRs MCD spectra are presented in Table 1. 

**Figure 3:**
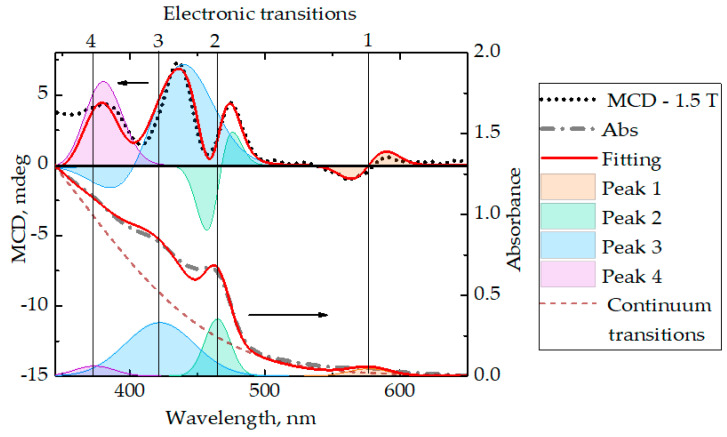
MCD spectrum fitting and absorption spectrum (bottom) of DiRs CdSe/CdS. On the MCD spectrum: the orange curve is the 1st band, the green curve is the 2nd band, the blue curve is the 3rd band, the violet curve is the 4th band, the dashed line is the spectrum of the MCD DiRs CdSe/CdS at a field strength of −1.5 T, the orange curve is an approximation.

Figure 3 shows the fitting of the MCD spectra DiRs CdSe/CdS. In the case of the MCD spectra of DiRs, the fitting was carried out in the range from 340 to 630 nm, X^2^ was 1.056. As in the case with QRs, asymmetry of the 2nd and the 3rd fitted bands indicates the contribution of both the ***A*** and ***B*** terms. The band related to the CdSe quantum dot contained in DiRs with a center at a wavelength of 577 nm has the shape of a Gaussian derivative, which indicates the presence of the ***A*** term without a noticeable contribution of the ***B*** term. The shortest wavelength band under consideration has a predominant contribution of the ***B*** term. The numerical values of the parameters ***A*** and ***B*** in the MCD spectra of DiRs are presented in Table 1. Table 1 shows the appearance of negative signs *B*_0_/*D*_0_. The signs of numerical values, according to the calculations, can be both positive and negative. They determine the sign of dichroism in a given transition induced in the positive direction of an external magnetic field.

Values of *A*_1_/*D*_0_ and *B*_0_/*D*_0_ for both QRs and DiRs (see Table 1) by the order of values are very similar to the corresponding parameters for CdSe nanoplates (where *A*_1_/*D*_0_~0.01–0.20 and *B*_0_/*D*_0_~0.0001–0.001 1/cm^−1^) [17]. QRs and DiRs, as well as nanoplates, demonstrate the relatively high values of the ***B*** term compared with CdSe QDs [16], where the contribution of the term ***B*** is negligible. The existing ***B*** term in elongated rods and planar nanoplatelets can be associated with the shape anisotropy of these nanostructures.

In the Dot-in-Rods, for the transition related to CdS QR within DiR, the ***B*** term is also observed in a magnitude comparable to the ***B*** terms in anisotropic particles, while in the CdSe QD contained in DiR only the ***A*** term is observed. According to the results in Table 1 the term ***A*** is present in almost all transitions, which means that all these transitions are degenerate, except perhaps the second and fourth transitions in the QRs.

In the case of QDs, by virtue of symmetry, all allowed transitions are triply degenerate, which leads to the contribution of the term ***A*** in each transition or in each group of transitions of close energy [17]. Changing of nanocrystal shape from zero-dimensional (0D) QDs to one-dimensional nanorods (1D) removes triple degeneracy of transition. The nanorods retain the 2-fold degeneracy (xy-polarization, perpendicular to the QR’s long axis) and have non-degenerate transitions (z-polarization, along the long axis). Contribution of the ***B*** term can relate to both the xy transition (it can also contain the contribution of the ***A*** term) and the z transition (it cannot contain the contribution of the ***A*** term). It is impossible to distinguish between these two cases at this stage.

## 4. Conclusions

Thus, in this work, we have investigated the electronic transitions in CdSe/ZnS QRs and CdSe/CdS DiRs by MCD spectroscopy. The effective values of the parameters ***A*** and ***B***, characterizing the splitting and mixing of states for the absorption bands observed in the spectra have been determined. The numerical values have been found to be *A*_1_/*D*_0_~10^−2^–0.8 and *B*_0_/*D*_0_~10^−6^–10^−3^ 1/cm^−1^. We believe that this research will be useful for further understanding of the electronic and optical properties of anisotropic semiconducting nanostructures.

## Figures and Tables

**Table 1:** *A*_1_/*D*_0_ and *B*_0_/*D*_0_ for QRs and DiRs.

Transition	Transition Energy, eV	Peak Center, nm	*A*_1_/*D*_0_	*B*_0_/*D*_0_, 1/cm^−1^
CdSe/ZnS QRs				
1	2.00	617	0.143	0.0004
2	2.15	576	0.005	0.0004
3	2.47	502	0.765	−6.3 × 10^−5^
4	2.74	452	0.093	0.0029
CdSe/CdS DiRs				
1	2.15	577	0.031	<6 × 10^−6^
2	2.66	465	0.110	−0.0003
3	2.93	422	0.452	0.0007
4	3.31	374	0.116	0.0018

## Supplementary Materials

The following are available online at https://www.mdpi.com/2079-4991/10/6/1059/s1, Figure S1: Second derivative of the absorption spectrum (on top), absorption spectrum (bottom), fitting of the absorption spectrum of QRs CdSe/CdS: orange curve is the 1st band, green curve is the 2nd band, blue curve is the 3rd band, violet curve is the 4th band, dashed curve is the a background absorption of unresolved continuumlike transitions increasing towards shorter wavelengths, orange curve is the approximation, Figure S2: Second derivative of the absorption spectrum (on top), absorption spectrum (bottom), fitting of the absorption spectrum of DiRs CdSe/CdS: orange curve is the 1st band, green curve is the 2nd band, blue curve is the 3rd band, violet curve is the 4th band, dashed curve is the a background absorption of unresolved continuumlike transitions increasing towards shorter wavelengths, black curve is the approximation, Table S1: The positions of the bands, half widths (FWHM), the maximum optical density ($D_{m}$) CdSe/ZnS QRs bands, Table S2: The positions of the bands, half widths (FWHM), the maximum optical density ($D_{m}$) CdSe/CdS DiRs bands. Figure S3: SEM images of (a) QRs CdSe/ZnS; (b) DiRs CdSe/CdS.
