# Supplementary material for: Investigation of Magnetic Circular Dichroism Spectra of Semiconductor Quantum Rods and Quantum Dot-in-Rods
Source: Nanomaterials (Basel). 2020 May 30;10(6):1059. doi: 10.3390/nano10061059 (PMC7352828; doi:10.3390/nano10061059)
Supplement: Supplementary file 1 [file nanomaterials-10-01059-s001.pdf]

Article

Investigation of Magnetic Circular Dichroism Spectra of Semiconductor Quantum Rods and Quantum Dot-in-Rods

Farrukh Safin [1,*], Vladimir Maslov [1], Yulia Gromova [2], Ivan Korsakov [1], Ekaterina Kolesova [1], Aliaksei Dubavik [1], Sergei Cherevkov [1] and Yurii K. Gun'ko [2]

[1] School of Photonics, ITMO University, 197101 St. Petersburg, Russia; maslov04@bk.ru (V.M.); korsakovivan@yandex.ru (I.K.); e.p.kolesova@gmail.com (E.K.); adubavik@corp.ifmo.ru (A.D.); S.cherevkov@gmail.com (S.C.)

[2] School of Chemistry, Trinity College, Dublin 2 Dublin, Ireland; yulia.a.gromova@gmail.com (Y.G.); IGOUNKO@tcd.ie (Y.K.G.)

* Correspondence: farruhsafin@gmail.com

CdSe/ZnS Quantum Rods Absorption Spectrum Fitting Results

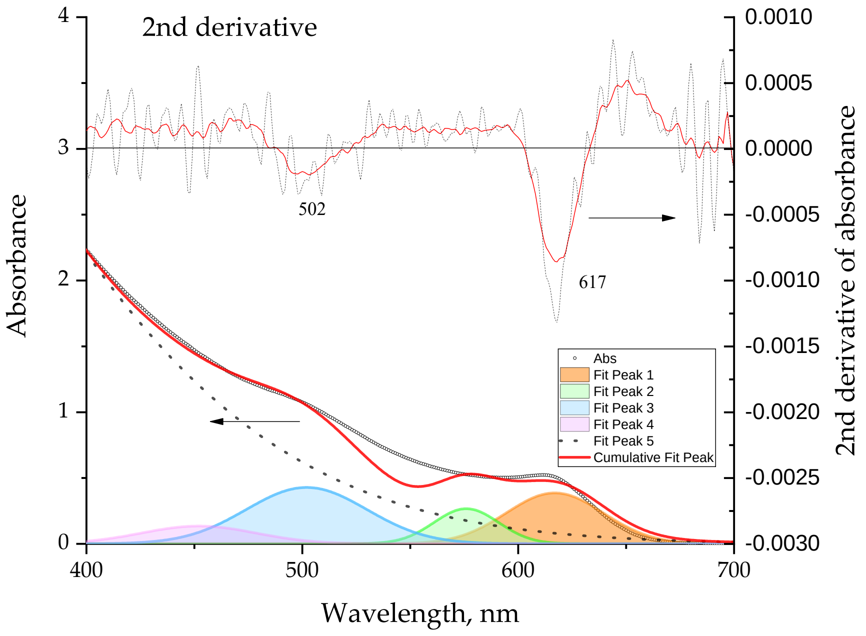

Figure S1. Second derivative of the absorption spectrum (on top), absorption spectrum (bottom), fitting of the absorption spectrum of QRs CdSe/CdS: orange curve is the 1st band, green curve is the 2nd band, blue curve is the 3rd band, violet curve is the 4th band, dashed curve is the a background absorption of unresolved continuumlike transitions increasing towards shorter wavelengths, orange curve is the approximation.

On the spectrum of the second derivative, 2 distinct bands can be seen at wavelengths of 617 nm and 502 nm. And also, two more less noticeable, but still distinguishable absorption bands with maxima at wavelengths of 576 nm band 452 nm. Table S1 presents the positions and widths bands of the QR CdSe/ZnS. The widths of the bands were determined from the fitting of the MCD spectra. The absorption spectra were fitted in the range from 400 to 700 nm; the X^2 value was 0.95.

Table S1. The positions of the bands, half widths (FWHM), the maximum optical density (D_m) CdSe/ZnS QRs bands

Transition	Energy, eV	Peak center, nm	FWHM, см$^{-1}$	Value D_m
1	2.00	617	1394	0.386
2	2.15	576	1066	0.266
3	2.47	502	2584	0.427
4	2.74	452	3069	0.134

CdSe/ZnS Dot-in-Rods Absorption Spectrum Fitting Results

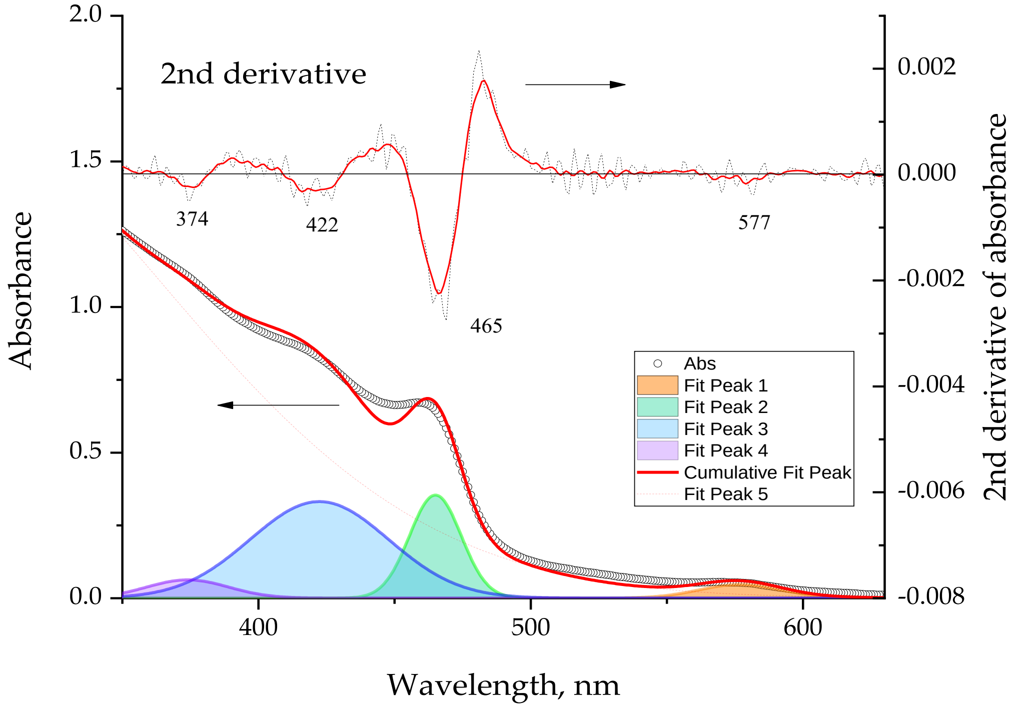

Figure 2. - Second derivative of the absorption spectrum (on top), absorption spectrum (bottom), fitting of the absorption spectrum of DiRs CdSe/CdS: orange curve is the 1st band, green curve is the 2nd band, blue curve is the 3rd band, violet curve is the 4th band, dashed curve is the a background absorption of unresolved continuumlike transitions increasing towards shorter wavelengths, black curve is the approximation.

Figure S2 shows the second derivative of the absorption spectrum of DiRs CdSe/CdS, from which the positions of the absorption bands at wavelengths of 577 nm, 465 nm, 422 nm, 374 nm can be seen. For DiRs, the fitting of the absorption spectra was carried out in the range from 340 nm to 630 nm, X^2 was 0.83. The bandwidths, as in the case of QR, were determined from the fitting of the MCD spectra DiRs CdSe/CdS. The positions and bandwidths are shown in Table S2.

Table S2. The positions of the bands, half widths (FWHM), the maximum optical density (D_m) CdSe/CdS DiRs bands

Transition	Energy, eV	Peak center, nm	FWHM, см$^{-1}$	Value D_m
1	2.15	577	1056	0.047
2	2.66	465	1035	0.354

3	2.93	422	3389	0.331
4	3.31	374	2531	0.145

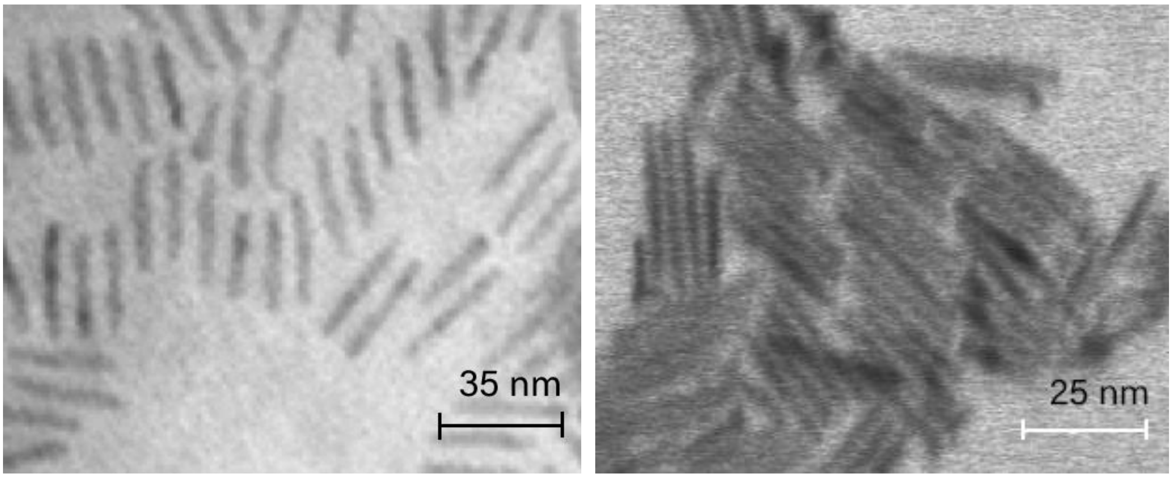

a. b

Figure S3. – SEM images of (a) QRs CdSe/ZnS; (b) DiRs CdSe/CdS.

SEM images were taken by scanning electronic microscope «Merlin Carl Zeiss».
